# Reaction or infection: topical chloramphenicol treatment

**DOI:** 10.1308/003588413X13511609955418

**Published:** 2013-01

**Authors:** RJ Livingston, JW Butterworth, P Belt

**Affiliations:** ^1^Greenslopes Hospital,Australia; ^2^Cairns Base Hospital,Australia

**Keywords:** Chloramphenicol, Basal cell carcinoma, Plastic surgery, Contact dermatitis, Hypersensitivity reaction

## Abstract

Chloramphenicol is a topical treatment that is used widely, especially in wounds around the eyes. In our practice there have been a number of cases of delayed hypersensitivity to chloramphenicol that has been mismanaged initially as an infective cellulitis. We hope to share some of our experience of this uncommon reaction to highlight the delayed reaction that can occur with topical application of this drug.

Chloramphenicol is a widely used topical ointment applied routinely to suture lines and skin grafts, particularly those on the face and around the eyes. Historically, chloramphenicol has attracted ‘bad press’ after being associated with anaphylaxis,^1^ blood dyscrasias, acute leukaemia, aplastic anaemia, hepatitis^2^ and contact dermatitis.^3–5^ Several of these reported associations following topical application of chloramphenicol (such as with leukaemia and aplastic anaemia) have been contested by controlled trials.^6,7^ In recent months, several of our patients have suffered cases of dermatitis following chloramphenicol application, which, being mistaken initially for wound infections, has had potentially deleterious health consequences.

Although well documented in the literature, dermatitis secondary to chloramphenicol is not well documented in the plastic surgery journals and its delayed presentation gives it particular relevance in this clinical setting.

It must be noted that neither of the patients in the cases described below were known to have any previous drug allergies.

## Case 1

The first case was a 69-year-old woman who underwent excision and Z-plasty repair of a basal cell carcinoma located in the inner canthus. Unfortunately, the margins were incomplete and she had to have the area re-excised to achieve adequate clearance. Over a 30-day period from her first excision to following the second excision, she applied chloramphenicol ointment routinely with no apparent adverse affect.

Despite a prophylactic course of antibiotics, the patient was readmitted to hospital on the seventh postoperative day after her re-excision with worsening erythema, swelling around the operative site and conjunctival injection. Clinically, she was diagnosed with an infection and started on intravenous flucloxacillin and penicillin while being continued on the topical therapy. After 48 hours without clinical improvement, her antibiotics were changed to vancomycin, which induced a ‘red man’ reaction and was subsequently ceased. Throughout her admission, she was apyrexial with normal inflammatory blood markers. Viral and bacterial swabs from the operative site returned showing no growth. After careful consideration and in consultation with the infectious disease team, all antibiotics were stopped and the chloramphenicol was withheld. The following day her perioperative erythema and swelling had improved markedly and she was discharged from hospital.

A review by the infectious disease specialists concluded the patient’s symptoms had been consistent with a contact dermatitis secondary to chloramphenicol ointment. This diagnosis was supported by consistently normal inflammatory markers, lack of fever and the resolution of symptoms following cessation of chloramphenicol. Interestingly, it later transpired that her daughter had also suffered adverse reactions to chloramphenicol.

## Case 2

**Figure 1:**
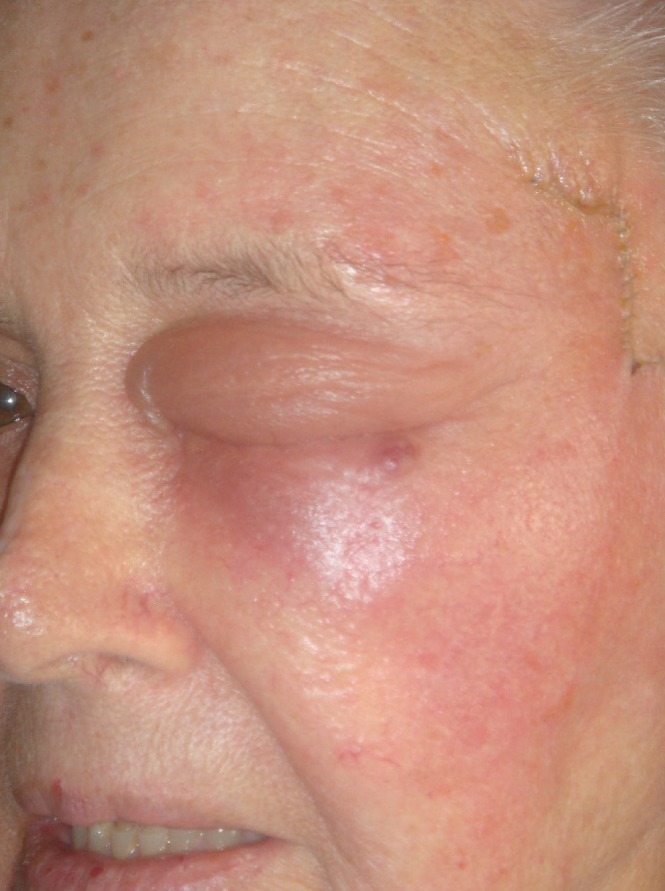
Case 2: photograph taken 24–48 hours following excision of basal cell carcinoma

Our second case was a 74-year-old woman who had had multiple skin excisions under our team between 2007 and 2009, receiving a total of 6 weeks of topical chloramphenicol therapy following each excision. Her most recent operation in 2009 involved excision of a basal cell carcinoma from her left temple and reconstruction with a bilateral advancement flap. Despite a course of prophylactic antibiotics, the wound became red, hot and swollen 24–48 hours following the procedure (Fig 1). The patient felt that these symptoms were due to the topical ointment and ceased its application without our knowledge. She was treated with oral antibiotics at this time for a suspected cellulitis and her symptoms resolved clinically over the next few days.

At the time of suture removal, under the advice of the practise nurse, topical chloramphenicol was recommenced. On application of the ointment, blepharal oedema and redness over the area became immediately apparent and so the medication was ceased. These symptoms again settled over the next 24 hours, confirming this was an adverse reaction.

## Discussion

The above patients are two recent cases among many seen previously in our practice presenting with delayed onset dermatitis in the setting of previously uncomplicated chloramphenicol use.

In susceptible individuals chloramphenicol has been shown to exhibit a delayed type hypersensitivity reaction.^8^ This type of reaction is the same as the reaction first demonstrated by Robert Koch in 1882 with his tubercle bacillus. The nature of the delayed type hypersensitivity reaction is that it has a postponed onset of 24–72 hours. This time lapse makes clinical differentiation from an infective origin all the more challenging.

The delayed onset of these hypersensitivity reactions misled our team initially in both the above cases, having potentially serious health consequences. Only following failed antibiotic treatment was an allergic reaction considered. Our differential diagnosis was further misled by the fact that both patients had used chloramphenicol ointment previously without clinical adverse effects.

Neither of our patients had evidence of systemic infection. The laboratory tests did not show raised C-reactive protein levels or leucocytosis and both patients remained apyrexial. However, both pyrexia and raised inflammatory markers may be present in patients with delayed type hypersensitivity reactions, thereby limiting their usefulness in delineating this differential diagnosis.

## Conclusions

Surgeons must maintain a high index of clinical suspicion for a chloramphenicol allergy, particularly in those patients who have developed perioperative site erythema and swelling within the first 24–72 hours of chloramphenicol application and do not respond to antibiotics. A delayed type hypersensitivity reaction must remain a prominent differential diagnosis even if symptoms occur outside these parameters or indeed if chloramphenicol has been used previously without adverse reactions. On consideration of these cases, we have adjusted our practice and now cease topical ointments prior to initiating systemic therapy for suspected infection.
